# Bracelet-Like Ni_0.4_Cu_0.6_O Microstructure Composed of Well-Aligned Nanoplatelets as a Superior Catalyst to the Hydrolysis of Ammonia Borane

**DOI:** 10.3389/fchem.2019.00776

**Published:** 2019-11-14

**Authors:** Xianfeng Li, Liucheng Gui, Huahong Zou

**Affiliations:** ^1^School of Chemistry and Materials Engineering, Huizhou University, Huizhou, China; ^2^State Key Laboratory for Chemistry and Molecular Engineering of Medicinal Resources, School of Chemistry and Pharmacy, Guangxi Normal University, Guilin, China

**Keywords:** heterogeneous catalysis, nanoplatelets, hydrogen production, ammonia borane, hydrolysis

## Abstract

The development of novel catalysts with both high catalytic activity and low cost toward the hydrolysis of ammonia borane is an important subject in the field of hydrogen energy. In this communications, Ni_x_Cu_1−x_O microstructures with different morphology have been synthesized and their catalytic activities in AB hydrolysis is studied. It's found that bracelet-like nanoplatelets were obtained at x = 0.4 and exhibit highest catalytic performance with turnover frequency of 33.43 mol_hydrogen_ min^−1^ ${\text{mol}}_{\text{cat}}^{-1}$, which much higher than those of most of CuNi-based catalysts in the literature. Pronounced synergistic effects between CuO and NiO in AB hydrolysis also have been observed. Due to the superior catalytic performance and cheapness, the prepared bracelet-like nanoplatelets Ni_0.4_Cu_0.6_O catalysts can be a strong catalyst candidate in AB hydrolysis.

## Introduction

Hydrogen is clean energy resource that has attracted extensive attention in the word. It can be regarded as one of the most promising green energy by which we can replace the traditional fossil fuel in view of the high calorific value, zero-emission, as well as renewable characteristics (Liu et al., 2010; Yang et al., 2010). However, how to store and produce hydrogen in safe and efficient way is still big problem in industrial scale applications. So far, many strategies have been developed for dealing with hydrogen storages and production. Among them, hydrolytic dehydrogenation of ammonia borane (AB) by a catalytic process has been deemed as an effective and reliable route (Wang et al., 2016; Du et al., 2017; Men et al., 2018). In this route, the proper catalyst is indispensable due to the slow kinetics of AB hydrolysis in the absence of a catalyst. In the literature, there are two typical types of catalysts toward AB hydrolysis, including noble-metal-based metals and alloys [for examples, Pt (Aijaz et al., 2012), Pd (Xi et al., 2012), Ru (Basu et al., 2009), and their alloy (Amali et al., 2013; Zhou and Xu, 2016)] and cheap metals and alloys [for examples, Co (Yan et al., 2010), Cu (Yao et al., 2014), and their alloys (Li et al., 2015; Bulut et al., 2016; Lu et al., 2018)]. Considering the low cost, the second types of catalysts seem to be more attractive in the practical applications. However, the activity of cheap metals and alloys in AB hydrolysis is far from satisfactory. In this regard, catalysts with both low cost and high catalytic activities are still highly desirable.

In this work, a series of nanostructures Ni_x_Cu_1−x_O catalysts and their activity in AB hydrolysis have been synthesized and studied. As far as we know, these catalysts used in AB hydrolysis have not been documented in the literature. It's found that the highest catalytic performance can be achieved at x = 0.4. In addition, the pronounced synergistic effects between CuO and NiO in AB hydrolysis have been observed. The findings in the present study can provide insight to help other researchers design inexpensive and highly active catalysts.

## Results and Discussion

**Figure 1:**
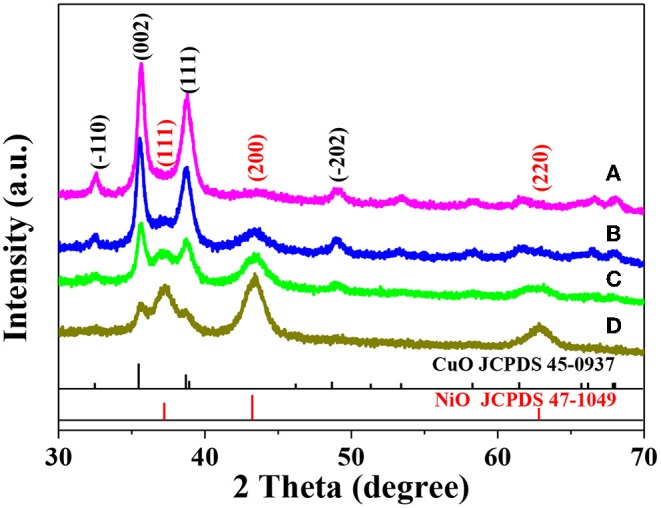
XRD patterns of Ni_0.2_Cu_0.8_O **(A)**, Ni_0.4_Cu_0.6_O **(B)**, Ni_0.6_Cu_0.4_O **(C)**, and Ni_0.8_Cu_0.2_O **(D)**.

Figure 1 shows the XRD patterns of different Ni_x_Cu_1−x_O catalysts. As can be seen, the characteristic peaks of both CuO and NiO are observed in these four XRD patterns. Evidently, as x increase, the intensity of peaks related to NiO increase while that of CuO decrease. Notably, it is difficult for us to judge whether the Ni_x_Cu_1−x_O is a homogeneous hybrid or just a mixture of CuO and NiO based on the XRD results alone. The XRD patterns of single-component CuO and NiO are shown in Figures S1a and b, respectively. Evidently, the characteristic peaks well match those of standard XRD patterns.

**Figure 2:**
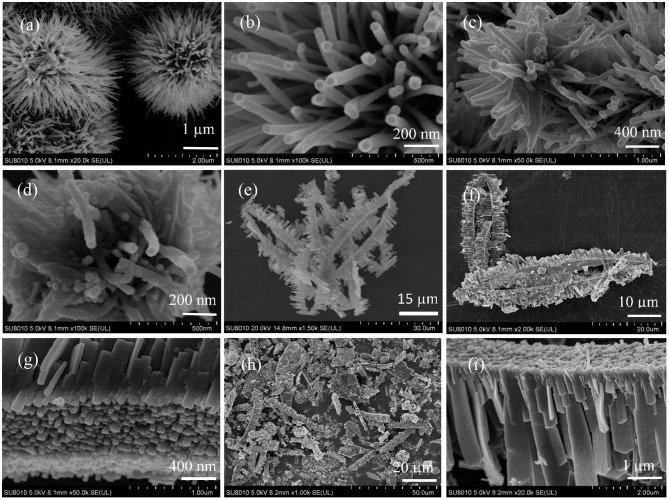
SEM images of Ni_0.8_Cu_0.2_O **(a,b)**, Ni_0.6_Cu_0.4_O **(c,d)**, Ni_0.4_Cu_0.6_O **(e–g)**, and Ni_0.2_Cu_0.8_O **(h,i)**.

**Figure 3:**
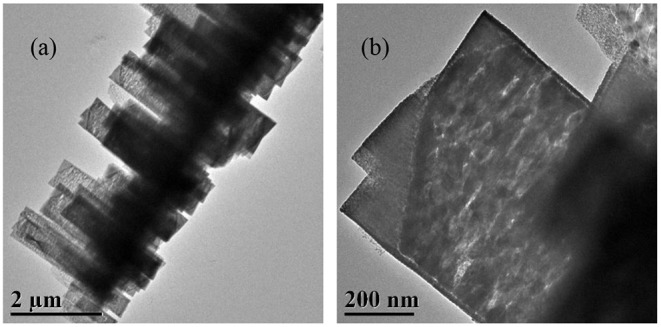
TEM images of Ni_0.4_Cu_0.6_O catalyst with low **(a)** and high **(b)** magnification.

All the Ni_x_Cu_1−x_O catalysts are analyzed with SEM and the results are shown in Figure 2. Figure 2a indicates that Ni_0.8_Cu_0.2_O is microspheres composed of nanowires with irradiation arrangement. The diameter of the nanowires is about 30 nm and length is 500–1000 nm. The morphology of Ni_0.6_Cu_0.4_O is very similar to that of Ni_0.8_Cu_0.2_O. Interestingly, the morphology of the products changes remarkably when x = 0.4. As shown in Figure 2e, a lot of belt-like agglomeration can be observed, some of which curls like rings. The typical width of the belts is about 2–4 μm. Figure 2g clearly indicates that these belts are composed of numerous well-arrayed nanoplatelets with the thickness of around 30 nm. The morphology of the Ni_0.2_Cu_0.8_O is similar to that of Ni_0.4_Cu_0.6_O, but the sizes of the small nanoplatelets become larger. The SEM images of CuO and NiO are displayed in Figure S2. CuO is the aggregation of micro-sized nanoplates and NiO is microspheres composed of nanosheets with thickness of about 30 nm. Figure 3a shows the TEM images of a part of belt-like agglomeration, confirming that the agglomeration is consisting of plenteous small nanoplatelets. Figure 3b indicates that the width of these nanoplatelets is about 500 nm. Notably, we carried out an ultrasonication treatment of the Ni_0.4_Cu_0.6_O rings and found that the ultrasonication treatment failed to break the rings into separated nanoplatelets. This finding implied that the Ni_0.4_Cu_0.6_O rings are integrative, not consisting of loosely connected nanoplatelets.

**Figure 4:**
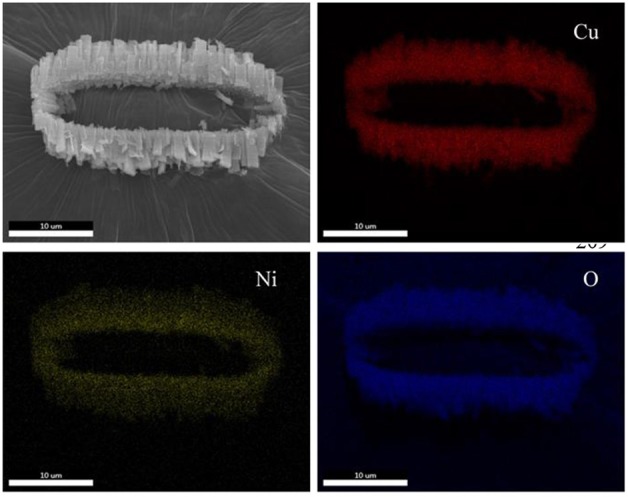
Elemental mapping of Ni_0.4_Cu_0.6_O catalyst.

Figure 4 displays the SEM image of a belt-like agglomeration and corresponding Cu, Ni, and O mapping. Interestingly, element of Cu, Ni, and O have almost the same mapping, indicating that these elements are uniformly distributed. This observation hints that our Ni_0.4_Cu_0.6_O catalyst is a homogeneous hybrid rather than a mixture of CuO and NiO.

**Figure 5:**
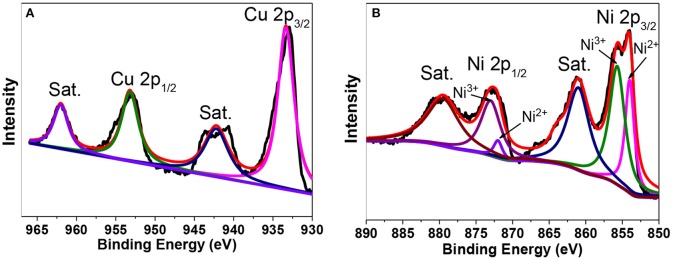
XPS spectra of Cu 2p **(A)** and Ni 2p **(B)**.

The XPS spectra of the Ni_0.6_Cu_0.4_O are shown in Figure 5. Figure 5A is XPS spectrum of the Cu in the 2p region. The peak at 933.2 and 953.3 eV are attributed to the binging energies of Cu 2p 3/2 and Cu 1/2 levels. The splitting of two peaks 20.1 eV, along with their position establishes the presence of CuO (Jana et al., 2010). In Figure 5B, the Ni 2p peaks can be decomposed into four peaks at 853.8, 855.6, 871.9, and 872.9 eV, besides the two satellite (Sat.) peaks. The first and third peaks can be ascribed to Ni^2+^ and the second and fourth peak can be assigned to Ni^3+^ (Zhao et al., 2009). Notably, although Ni^3+^ has been detected in the surface of the Ni_0.6_Cu_0.4_O sample, no characteristic peaks of Ni_2_O_3_ has been observed in the XRD pattern. This suggests that there is only a small amount of Ni^3+^ in the surface of the sample. This is understandable because our sample has been calcined during the synthesis, in which the oxidative transformation of Ni^2+^ to Ni^3+^ maybe happen on the surface.

**Figure 6:**
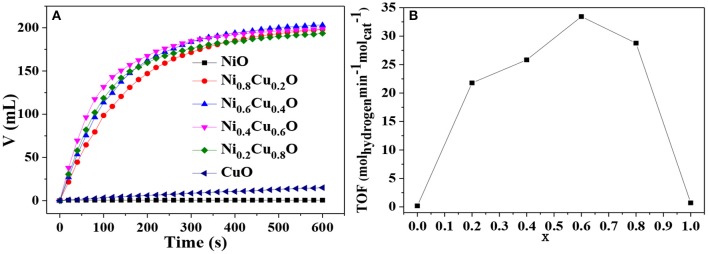
**(A)** Hydrogen release from AB solution in the presence of different catalyst and **(B)** TOF values vs. x in Ni_x_Cu_1−x_O.

Figure 6A shows the hydrogen evolution from AB solution in the presence of different catalysts. When NiO acts as a catalyst, no hydrogen is released from AB solution, indicating that NiO is inactive to AB hydrolysis. When CuO is used instead, the hydrogen release is quite slow, demonstrating that CuO possesses low catalytic activity. Interestingly, when the Ni_x_Cu_1−x_O microstructures serve as catalysts, hydrogen will be produced constantly and fast until the hydrolytic reaction complete. Figure 6B shows the TOF for different catalysts. It is found that Ni_0.4_Cu_0.6_O exhibit the highest TOF of 33.43 mol_hydrogen_ min^−1^ ${\text{mol}}_{\text{cat}}^{-1}$. In contrast to the CuNi based catalysts in the literature, our Ni_0.4_Cu_0.6_O catalyst exhibit significantly improved catalytic activity. This value is higher than that of Cu/RGO (3.61 mol_hydrogen_ min^−1^ ${\text{mol}}_{\text{cat}}^{-1}$) (Yang et al., 2014), nanoporous nickel spheres (19.6 mol_hydrogen_ min^−1^ ${\text{mol}}_{\text{cat}}^{-1}$) (Cao et al., 2010). It is also higher than that of Cu_2_Ni_1_@MIL-101 (20.9 mol_hydrogen_ min^−1^ ${\text{mol}}_{\text{cat}}^{-1}$) (Gao et al., 2018), CuNi/MCM-41 (15.0 mol_hydrogen_ min^−1^ ${\text{mol}}_{\text{cat}}^{-1}$) (Lu et al., 2014), and CuNi@carbon (0.2 mol_hydrogen_ min^−1^ ${\text{mol}}_{\text{cat}}^{-1}$) (Yousef et al., 2012). However, it is still slightly lower than that of CuNi-CeO_2_/graphene oxide recently reported in the literature (34.4 mol_hydrogen_ min^−1^ ${\text{mol}}_{\text{cat}}^{-1}$) (Zhou et al., 2017). Notably, the TOF value for Ni_*x*_Cu_1−*x*_O is significantly higher than the sum of those for NiO and CuO. This observation hints that there is significant synergistic effect between NiO and CuO in AB hydrolysis. There are two possible reasons for this. Firstly, according to Liao et al. (2018) and Lu et al. (2019), the real active species of oxide-based catalysts is the metals or alloys formed by the reduction of oxides by AB. However, it is difficult to reduce CoO and NiO to their metallic states on account of their low reduction potential (Ni^2+^/Ni: −0.257 V vs. SHE; Co^2+^/Co: −0.280 V vs. SHE). When CuO is present, AB can easily reduce CuO to Cu due to the high reduction potential (Cu^2+^/Cu: 0.337 V vs. SHE), which is favorable for the reduction of CoO and NiO. Thus, active species can be formed immediately. Secondly, the modification of the surface electronic structure and chemical properties of the nanoalloy through the strain and ligand effects between two metals can synergistically improve the catalytic activity (Liao et al., 2018). Notably, our Ni_0.6_Cu_0.4_O catalysts is more active than CuNi alloys. As mentioned above, CuNi alloys will be *in-situ* formed on the surface of Ni_0.6_Cu_0.4_O catalysts. The unreacted Ni_0.6_Cu_0.4_O will act as a support. Thus, there is a metal-support effect within our catalyst. It is possible that the metal-support effect plays an important role in determining their high catalytic activity.

**Figure 7:**
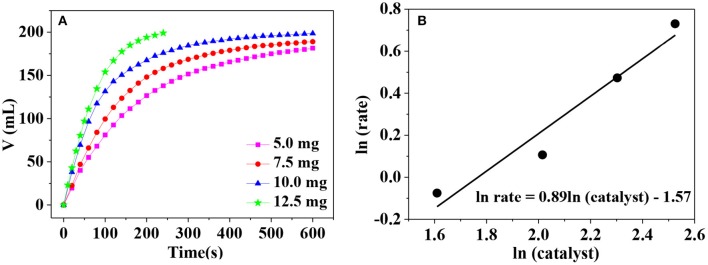
**(A)** Hydrogen release from AB solution at different catalyst dosage; **(B)** the relationship between logarithm of mass of catalyst and catalytic rate.

To investigate the amount of catalyst on the hydrogen generation rate, catalytic hydrolysis of AB catalyzed by different catalyst dosage is carried out. As can be seen in Figure 7A, the hydrogen generation rate becomes larger and larger when the catalyst dosage increases in the range from 5.0 to 12.5 mg. This is understandable because more active sites are provided for the adsorption of AB in the presence of larger catalyst dosage. To further clarify the relationship between the hydrogen generation rate (*r*) and the catalyst dosage (*m*), ln*r* vs. ln*m* was plotted in Figure 7B. The slope of the fitted line is 0.89, very close to 1, indicating that AB hydrolysis is a pseudo first-order reaction with respect to the catalyst. Thus, the hydrogen generation rate can be easily controlled by adjusting the catalyst dosage in the practical applications.

**Figure 8:**
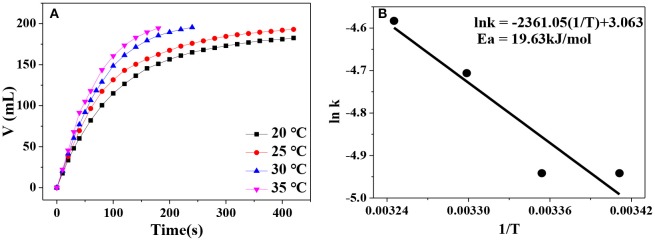
**(A)** Hydrogen evolution at various catalytic temperature in the range of 20–35°C; **(B)** the relationship between logarithm of k and 1/T.

Besides the hydrogen generation rate, apparent activation energy is another important parameter that can be applied to roughly assess the catalytic activity of a catalyst. In general, an active catalyst can significantly lower the reaction energy barrier (apparent activation energy) and thus increase the reaction rate. Correspondingly, low apparent activation energy of a catalytic reaction reflects that the catalyst possesses high catalytic activity. In Figure 8A, hydrogen evolution curves at different reaction temperature are displayed. Obviously, higher reaction temperature leads to faster hydrogen generation rate. By plotting lnk vs. ln(1/T), a fitted straight line is generated. According to the Arrhenius equation, the apparent activation energy of AB hydrolysis for our Ni_0.4_Cu_0.6_O catalyst is calculated to be about 19.63 kJ/mol (Figure 8B), which is lower than that of CuNi/MCM-41 (38 kJ/mol) (Lu et al., 2014) and Cu_2_Ni_1_@MIL-101 (32.2 kJ/mol) (Gao et al., 2018).

**Figure 9:**
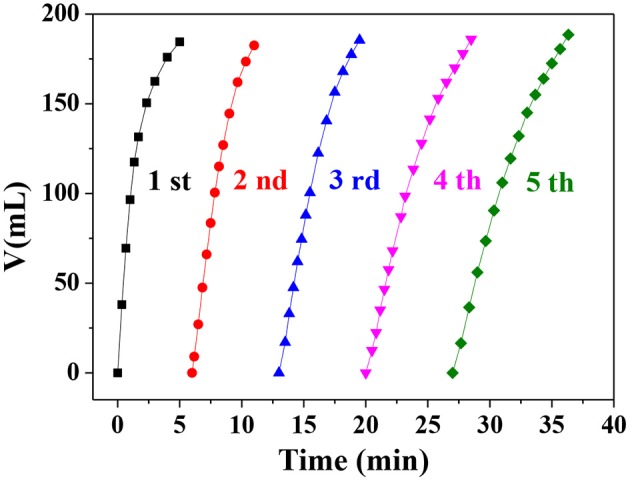
The hydrogen evolution at various catalytic cycles.

To investigate the stability and reusability of the Ni_0.4_Cu_0.6_O catalyst, the catalytic hydrolysis reaction was carried out repeatedly. As shown in Figure 9, the molar ratio of hydrogen to AB is 3 at the fifth catalytic run, verifying that the 100% hydrogen release ratio can be achieved. However, the catalyst activity of the catalyst decreases slightly after 5 catalytic runs. The SEM image of the used catalyst in Figure S3 indicates that the morphology of used catalyst remains almost unchanged. These findings demonstrate Ni_0.4_Cu_0.6_O catalyst have good reusability and relatively high stability.

## Conclusion

In summary, nanostructured Ni_x_Cu_1−x_O cat with different morphology have been prepared and their catalytic activity in AB hydrolysis has been studied. It is found that the belt-like Ni_0.4_Cu_0.6_O composed of plenteous well-aligned nanoplatelet exhibit the highest catalytic performance with turnover frequency of 33.43 mol_hydrogen_ min^−1^ ${\text{mol}}_{\text{cat}}^{-1}$, which is much higher than that of most of CuNi-based catalysts in the literature. It is interesting to note that there is pronounced synergistic effect between CuO and NiO in AB hydrolysis. Owing to its superior catalytic performance and cheapness, the Ni_0.4_Cu_0.6_O catalysts can be strong catalyst candidate in AB hydrolysis.

### Synthesis

All of the reagents are of analytic grade. In a typical process, CuSO_4_·5H_2_O and NiSO_4_·6H_2_O with a total metal ions of 5.0 mmol were dissolved in 20 mL water to form solution A. Twenty millimole urea was dissolved in 20 mL water to form solution B and 1.0 g cetyltrimethylammonium bromide (CTAB) was dissolved in 40 mL water to form solution C. After solution A and B were mixed, solution C was added to the mixture solution under stirring. After that, the resulted solution was poured in to a Teflon-lined autoclave, which was sealed and subjected to a heat-treatment at 160°C for 10 h. The solid product obtained at the bottom of the Teflon-lined autoclave was washed and calcined at 400°C for 4 h.

### Characterizations

Rigaku D/Max-1200X diffractometer with Cu Kα radiation was applied to record the X-ray powder diffraction (XRD) patterns of the samples. Hitachi Su-8010 field emission scanning electron microscope (FE-SEM) was used to analyze the morphology of the samples. Elemental mapping analysis was carried out using an EDAX Octane Elite energy disperse spectrometer (EDS) coupled with FE-SEM. Transmission electron microscopy (TEM) and high-resolution TEM (HRTEM) images were obtained on a FEI Tecnai G2 F20 S-TWIN transmission electron microscope. X-ray photoelectron spectroscopy (XPS) was performed on a Kratos Axis Ultra DLD X-ray photoelectron spectrometer with Al Kα radiation.

### Catalytic Experiments

In a typical experiment, catalyst with weight of 10.0 mg was dispersed into 10.0 mL water under ultrasonication. Then, 10 mL mixed solution of AB (0.3 M) and NaOH (2 M) was added into the vessel, which was sealed and connected to a glass burette. NaOH was used in the catalytic process because it could enhance AB hydrolysis (Yan et al., 2016). The reaction vessel was immersed into a water bath at temperature of 298 K. The volume of the produced hydrogen was determined by recording the displacement of water in the gas burette.

## Data Availability Statement

The datasets generated for this study are available on request to the corresponding author.

## Author Contributions

Synthesis of the sample, writing-original draft preparation, and investigation of the catalytic performance were performed by XL. Characterization and analysis of the sample by XL and LG. Supervision, funding acquisition and writing-review, and editing by XL and HZ. All authors have given their approval to the final version of the manuscript.

### Conflict of Interest

The authors declare that the research was conducted in the absence of any commercial or financial relationships that could be construed as a potential conflict of interest.
