# Supplementary material for: Bracelet-Like Ni0.4Cu0.6O Microstructure Composed of Well-Aligned Nanoplatelets as a Superior Catalyst to the Hydrolysis of Ammonia Borane
Source: Front Chem. 2019 Nov 14;7:776. doi: 10.3389/fchem.2019.00776 (PMC6868442; doi:10.3389/fchem.2019.00776)
Supplement: Supplementary file 1 [file Table_1.DOCX]

**Bracelet-like Ni_0.4_Cu_0.6_O microstructure composed of well-aligned nanoplatelets as a superior catalyst to the hydrolysis of ammonia borane**

**Xianfeng Li,^[1]^ Liucheng Gui,^[2]^ Huahong Zou^[2]^**

^1^School of chemistry and Materials Engineering, Huizhou University, Huizhou 516007, China.

^2^State Key Laboratory for Chemistry and Molecular Engineering of Medicinal Resources, School of Chemistry & Pharmacy, Guangxi Normal University, Guilin 541004, P. R. China.

***Correspondence:**Corresponding Author: Xianfeng Li and Liucheng Gui

[Email](mailto:Email): [wind9425@163.com](mailto:wind9425@163.com), guiliucheng2000@163.com

Figure S1 XRD patterns of CuO (b) and NiO (b).


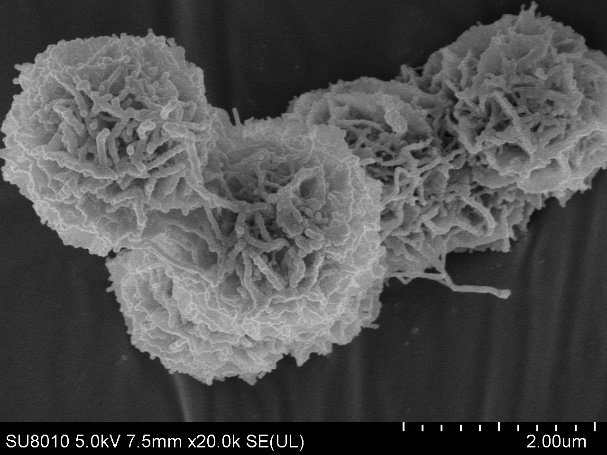


**(b)**

**(d)**

**(a)**

**(c)**


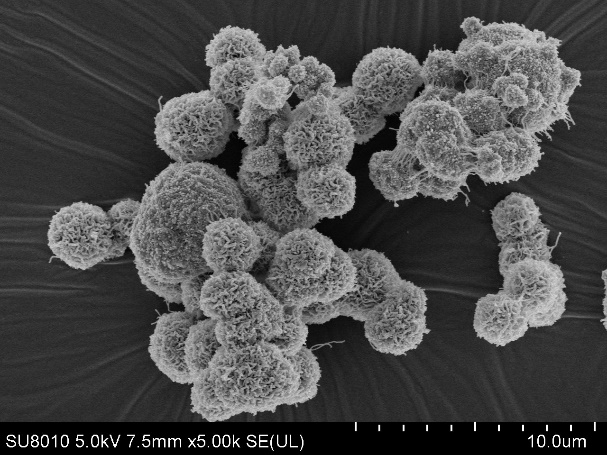

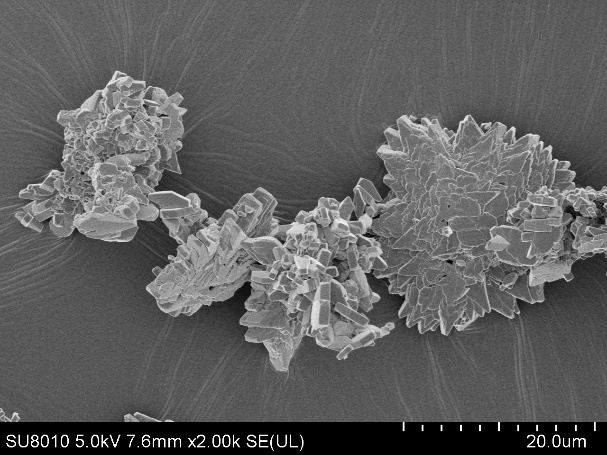

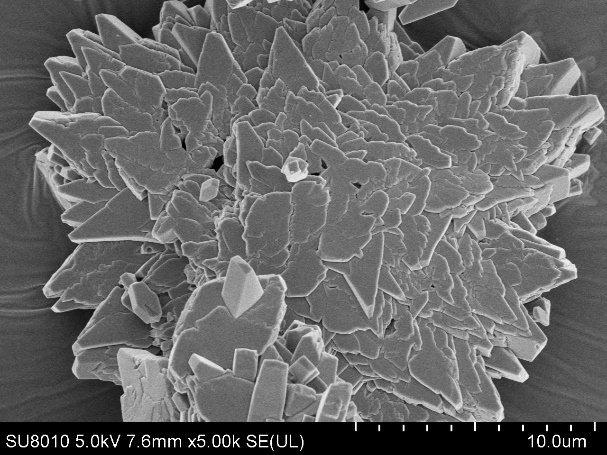


Figure S2 SEM images of CuO (a, b) and NiO (c, d).


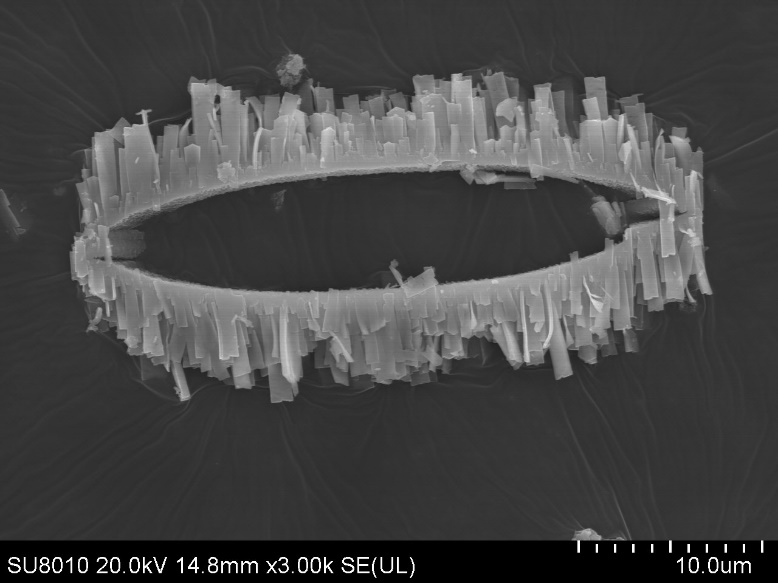


Figure S3 SEM image of the used Ni_0.4_Cu_0.6_O catalyst.
